# Direct Pharmacological Activation of AMPK Extends Lifespan in Yeast, Worms and Flies

**DOI:** 10.1111/acel.70721

**Published:** 2026-09-29

**Authors:** Eliano dos Santos, Marie Blickling, Fiona C. Leiper, John‐Patrick Alao, Claudia Lennicke, Andrea Foley, Jon R. Wilson, Steven J. Gamblin, Bénédicte Chazaud, Giovanna Lollo, Rémi Mounier, Gaëtan Juban, Charalampos Rallis, David Carling, Filipe Cabreiro, Helena M. Cochemé

**Affiliations:** ^1^ MRC Laboratory of Medical Sciences (LMS) London UK; ^2^ Institute of Clinical Sciences Imperial College London London UK; ^3^ CECAD Research Cluster University of Cologne Köln Germany; ^4^ School of Biological and Behavioural Sciences Queen Mary University of London London UK; ^5^ The Francis Crick Institute London UK; ^6^ Institut NeuroMyoGène, Physiopathologie et Génétique du Neurone et du Muscle, Université Claude Bernard Lyon 1, Inserm U1315, CNRS UMR 5261 Université de Lyon Lyon France; ^7^ Laboratoire d'Automatique, de Génie des Procédés et de Génie Pharmaceutique, Université Claude Bernard Lyon 1 CNRS UMR 5007 Villeurbanne France; ^8^ Institut Universitaire de France (IUF) Paris France; ^9^ Center for Molecular Medicine Cologne (CMMC) University of Cologne Köln Germany

**Keywords:** ageing, AMPK, *Caenorhabditis elegans*, *Drosophila melanogaster*, energy metabolism, lifespan, pharmacology, *Schizosaccharomyces pombe*

## Abstract

AMP‐activated protein kinase (AMPK) is a key evolutionarily conserved sensor of energy homeostasis and plays a central role in metabolic health and disease. AMPK has also been implicated in ageing; however, most in vivo drug studies rely on the use of indirect activators, such as metformin, which have complex modes‐of‐action, therefore making conclusions on the specific role of AMPK more challenging. Here, we demonstrate that direct activation of AMPK with the compound 991 extends lifespan in *Drosophila melanogaster*, *Caenorhabditis elegans* and *Schizosaccharomyces pombe*. In mice, 991 treatment induces a pro‐longevity proteomic profile, highlighting the potential for translation to mammals. Overall, our study provides important proof‐of‐principle for AMPK as a pharmacological target with longevity benefits.

## Graphical abstract

AMP‐activated protein kinase (AMPK) is a key evolutionarily conserved sensor of energy homeostasis, which is implicated in metabolic health and disease, as well as in ageing. Here, we show that direct pharmacological activation of AMPK with the compound 991 in vivo extends lifespan in multiple model organisms: the fission yeast *Schizosaccharomyces pombe*, the nematode worm *Caenorhabditis elegans* , and the fruit fly *Drosophila melanogaster*. In mice, 991 treatment induces a pro‐longevity proteomic profile, highlighting the potential for translation to mammals. Overall, our cross‐species study provides important proof‐of‐principle for AMPK as a pharmacological target with longevity benefits.

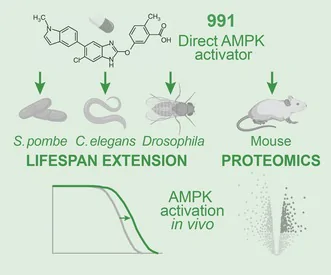

## 1 Introduction

AMP‐activated protein kinase (AMPK) is a fundamental sensor of metabolic status and stress, which is universal throughout eukaryotes. AMPK plays a central role in energy homeostasis and is therefore an attractive therapeutic target in health and disease (Carling 2017). AMPK has also been implicated as a regulator of longevity (Burkewitz et al. 2014, 2016). The role of AMPK in survival is supported by evidence from model organisms, including the fruit fly *Drosophila melanogaster* , the nematode worm *Caenorhabditis elegans* and the fission yeast *Schizosaccharomyces pombe*, where genetic mutants and/or dietary interventions resulting in AMPK activation are long‐lived (Apfeld et al. 2004; Mair et al. 2011; Stenesen et al. 2013; Ulgherait et al. 2014; Burkewitz et al. 2015; Rallis et al. 2017; Weir et al. 2017; Ling et al. 2020).

AMPK consists of a heterotrimer, with a catalytic α subunit and regulatory β and γ subunits, and displays strong evolutionary conservation across eukaryotes, as evident from sequence alignments between the human, fly, worm and yeast orthologues (Figure S1A,B). Maximal kinase activity of AMPK requires phosphorylation of a conserved threonine in the α subunit (T172 in human α2, T184 in *Drosophila*, T243 in *C. elegans* AAK‐2, and T189 in *S. pombe* Ssp2).

Many studies utilise pharmacological targeting of AMPK, for instance by the anti‐diabetic drug metformin, which activates AMPK indirectly by altering the cellular AMP/ADP:ATP ratio. Beyond its anti‐diabetic action, metformin treatment in humans also shows broad health benefits, protecting against age‐related diseases such as cancer (Pryor and Cabreiro 2015). Importantly, metformin extends lifespan in *C. elegans* (Onken and Driscoll 2010; Cabreiro et al. 2013; Pryor et al. 2019), *Drosophila* (Pryor et al. 2019) and mice (Anisimov et al. 2008; Martin‐Montalvo et al. 2013), and is currently the focus of planned geroprotective clinical trials in humans (Barzilai et al. 2016). However, metformin is an indirect activator of AMPK, affecting many other key metabolic pathways, including via mitochondrial and lysosomal targets (Pryor and Cabreiro 2015; Carling 2017; Ma et al. 2022; Bridges et al. 2023). Furthermore, its mode‐of‐action in vivo is complex, with studies in both worms and flies showing that the longevity effects of metformin on the host occur via the microbiota (Cabreiro et al. 2013; Pryor et al. 2019; dos Santos and Cochemé 2024), with evidence for bacterial involvement also in mammals (Pryor et al. 2020) (e.g., metformin is effective if administered orally, not intravenously).

To overcome the complexity and non‐specificity of metformin, studies with direct AMPK activators are essential to fully appreciate the therapeutic potential of AMPK in health and ageing. Here, we apply the direct AMPK activator 991 (Figure 1A) (Xiao et al. 2013) in yeast, worms, flies and mice to investigate its potential pro‐longevity effects.

**FIGURE 1:**
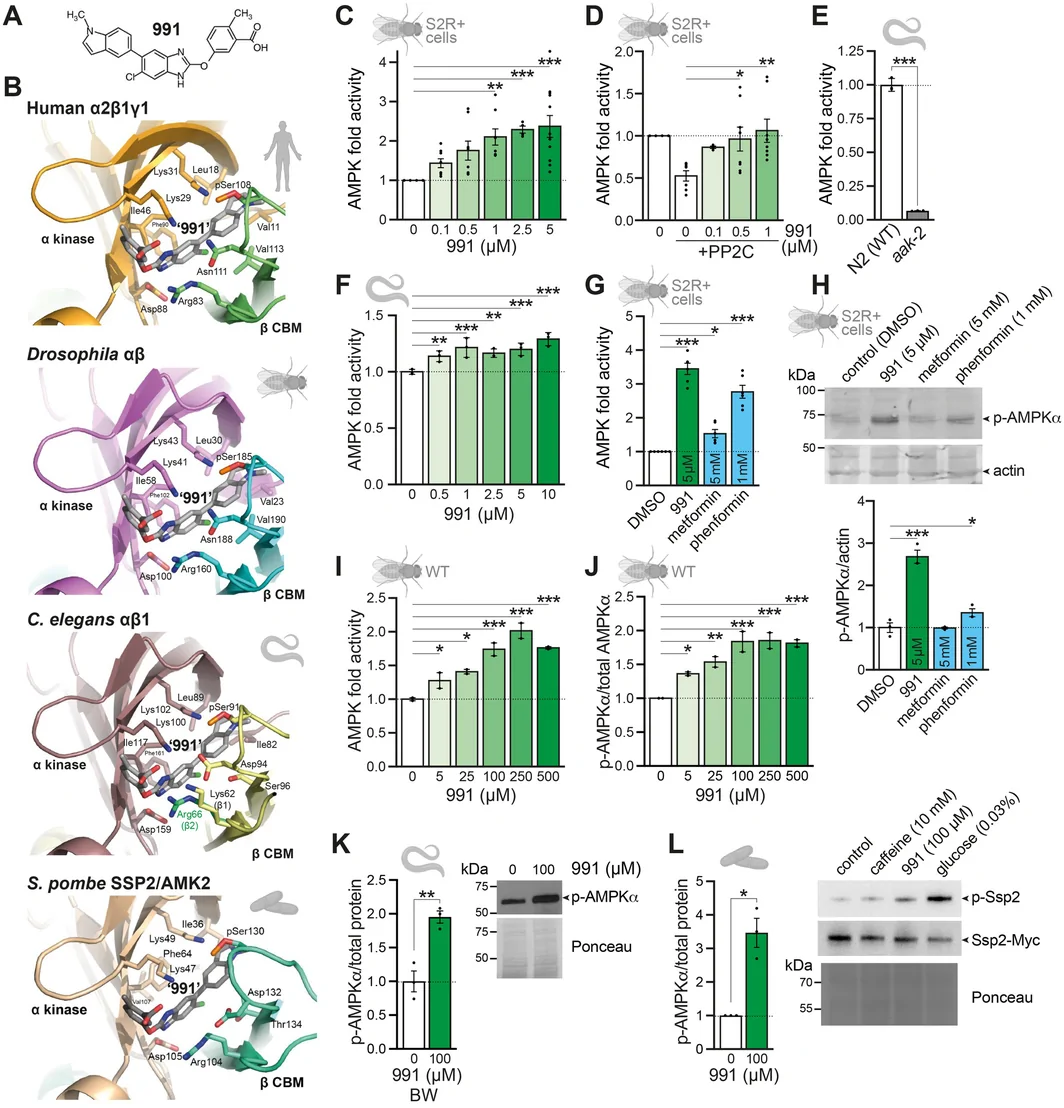
Direct pharmacological activation by 991 of AMPK from *Drosophila*, *C. elegans* and *S. pombe* in cell culture and in vivo. (A) Chemical structure of the 991 compound. (B) Crystal structure of human AMPK α2β1γ1 (PDB: 4CFE), and homology models of *Drosophila*, *C. elegans* (β1 and β2) and *S. pombe* AMPK. 991 binds between the interface of the α and β subunits. Key coordinating residues are highlighted. (C–F) In vitro activation of fly and worm AMPK by 991. (C) Allosteric activation by 991 of *Drosophila* AMPK, IP‐purified from S2R+ cells (*n* = 4–12 ± SEM; one‐way ANOVA against the 0 μM DMSO control: 0.1 μM, *p* = 0.2882; 0.5 μM, *p* = 0.0701; 1 μM, *p* = 0.0099; 2.5 μM, *p* = 0.0090; 5 μM, *p* = 9.57 × 10^−4^). (D) 991 protects fly S2R+ AMPK from dephosphorylation upon treatment with the phosphatase PP2C (*n* = 4–8 ± SEM; one‐way ANOVA against the 0 μM + PP2C control: 0.1 μM + PP2C, *p* = 0.1057; 0.5 μM + PP2C, *p* = 0.0152; 1 μM + PP2C, *p* = 0.0037). (E) Detection of AMPK activity in protein lysates extracted from WT (N2) worms but not AMPKα mutants (*aak‐2*) (*n* = 3 ± SD; *p* = 4.67 × 10^−6^, unpaired two‐tailed *t*‐test). (F) Allosteric activation of *C. elegans* AMPK in protein lysates extracted from WT (N2) worms (*n* = 3 ± SD; one‐way ANOVA against the 0 μM DMSO control: 0.5 μM, *p* = 0.0096; 1 μM, *p* = 4.50 × 10^−4^; 2.5 μM, *p* = 0.0032; 5 μM, *p* = 8.20 × 10^−4^; 10 μM, *p* = 3.16 × 10^−5^). (G, H) Activation of fly AMPK in cell culture, comparing 991 treatment with the indirect activators metformin and phenformin. (G) Kinase assay (*n* = 6 ± SEM; one‐way ANOVA against the DMSO control: 5 μM 991, *p* = 9.51 × 10^−11^; 5 mM metformin, *p* = 0.0150; 1 mM phenformin, *p* = 2.32 × 10^−8^). (H) Western blotting of phospho‐AMPKα normalised to Actin (*n* = 3 ± SEM; one‐way ANOVA against the DMSO control: 5 μM 991, *p* = 3.93 × 10^−6^; 5 mM metformin, *p* = 0.9581; 1 mM phenformin, *p* = 0.0491). (I, J) Activation of *Drosophila* AMPK by 991 in vivo, administered dietarily in defined medium for 7 days. (I) Kinase assay (*n* = 2 ± range; one‐way ANOVA against the 0 μM DMSO control: 5 μM, *p* = 0.0487; 25 μM, *p* = 0.0108; 100 μM, *p* = 5.85 × 10^−4^; 250 μM, *p* = 1.02 × 10^−4^; 500 μM, *p* = 4.93 × 10^−4^). (J) Western blotting of phospho‐AMPKα normalised to total AMPKα (*n* = 2 ± range, one‐way ANOVA against the 0 μM DMSO control: 5 μM, *p* = 0.0265; 25 μM, *p* = 0.0049; 100 μM, *p* = 5.24 × 10^−4^; 250 μM, *p* = 4.77 × 10^−4^; 500 μM, *p* = 6.15 × 10^−4^). (K) Activation of BW25113‐fed *C. elegans* AMPK by 991 in vivo based on western blotting of phospho‐AMPKα normalised to total protein (*n* = 3 ± SEM; *p* = 0.0061, unpaired two‐tailed *t*‐test). (L) Activation of *S. pombe* AMPK by 991 in vivo based on western blotting of phospho‐AMPKα normalised to total protein (*n* = 3 ± SEM; *p* = 0.0295, unpaired two‐tailed *t*‐test with Welch's correction). Treatment with caffeine (10 mM) or glucose (0.03%) are included as positive controls. *p < 0.05; **p < 0.01; ***p < 0.001.

## 2 Results

### 2.1 Direct Pharmacological Activation by 991 of AMPK From *Drosophila*, *C. elegans* and *S. pombe* in Cell Culture and In Vivo

So far, the compound 991 has been applied to mammalian systems; therefore, we first tested whether the action of 991 was evolutionarily conserved in yeast and invertebrate model organisms. As 991 binds to AMPK in a pocket at the interface of the α and β subunits (Xiao et al. 2013), known as the ‘ADaM’ (allosteric drug and metabolite) site (Steinberg and Carling 2019), we performed modelling of this region against the known human crystal structure (Xiao et al. 2011) (Figure 1B). The high amino acid sequence conservation of the AMPK α and β subunits (Figure S1A,B) translates to strong overlap of the predicted 991 binding pocket, including a key serine residue in AMPKβ (S108 in human, S185 in *Drosophila*, S91 in *C. elegans* , and S130 in *S. pombe*) known to interact with 991 (Xiao et al. 2013). AMPK activation by 991 requires the presence of the carbohydrate‐binding module (CBM) within the β subunit (Xiao et al. 2013). Interaction of the β subunit C‐interacting helix with the kinase αC helix is important for allosteric activation of AMPK (Carling 2017) and is stabilised by conserved hydrophobic residues, such as leucine L166 in the human β subunit (L243 in *Drosophila*, L149 in *C. elegans* , and I186 in *S. pombe*; Figure S1B).

Consistent with this structural modelling, we performed a kinase assay on AMPK purified by immunoprecipitation (IP) from a *Drosophila* cell line (S2R+) and showed that 991 was able to allosterically activate fly AMPK in a dose‐dependent manner (Figure 1C). As with mammalian AMPK, 991 binding also protected fly AMPK against dephosphorylation upon phosphatase treatment (Figure 1D). Similarly, we were able to detect AMPK activity from WT worm extracts, but not *aak‐2* mutants, null for the AMPKα orthologue (Figure 1E), and confirmed activation of worm AMPK by 991 in vitro (Figure 1F). The degree of allosteric activation by 991 depends on the isoform composition of the AMPK complex (Carling 2017). The preferred residue at the +3 position after S108 is an asparagine in human β1 (N111), which is also found in *Drosophila* (N188). In contrast, an aspartic acid is present in human β2 (D111) and the *C. elegans* orthologues (D94), which may explain why worm AMPK is more weakly activated by 991 than flies. Nevertheless, 991 activates both fly and worm AMPK.

Next, we compared the action of 991 against the indirect AMPK activators metformin and phenformin to assess their relative potency. Both by a kinase assay (Figure 1G) and western blotting against phospho‐AMPKα (Figure 1H, Figure S1C), we found that μM concentrations of 991 were able to activate fly AMPK, compared to mM concentrations of metformin and phenformin in *Drosophila* cell culture.

To administer 991 to flies in vivo, we utilised a chemically defined medium, shown to have improved drug bioavailability compared to a standard complex diet (Piper et al. 2014). Indeed, 991 activated fly AMPK dose‐dependently in vivo when fed via defined medium (Figure 1I), but not yeast‐based medium (Figure S1D), as assessed by a kinase assay. In vivo treatment with 991 activated AMPK in *Drosophila*, *C. elegans* and *S. pombe*, as determined by western blotting against phospho‐AMPKα (Figure 1J–L, Figure S1E–G). Therefore, the direct AMPK activator 991 is functional in flies, worms and yeast, allowing for the role of AMPK to be tested in the context of longevity within these tractable model systems.

### 2.2 991 Modulates Metabolism and Extends Lifespan in *Drosophila*, *C. elegans* and *S. pombe*

Having confirmed that 991 activated AMPK in an evolutionarily conserved manner, we next explored the effects on physiology and survival. AMPK plays an important role in energy homeostasis and fat metabolism (Carling 2017). We found that 991 decreased whole body triglyceride (TAG) levels dose‐dependently in the fly (Figure 2A), consistent with previous observations upon metformin treatment (Slack et al. 2012). We did not observe changes in glycogen content (Figure S2A). The decreased lipid reserves were not due to altered feeding, as 991 did not affect feeding rates in naïve 7 day old flies (Figure S2B), suggesting that the flies were not able to taste the drug, or after 7 days of treatment, indicating that 991 does not decrease food intake (Figure S2B,C). Similarly, 991 affected lipid metabolism in worms, as assessed by BODIPY staining (Figure 2B, Figure S2D). Therefore, in vivo administration of 991 exerts physiological effects in flies and worms.

**FIGURE 2:**
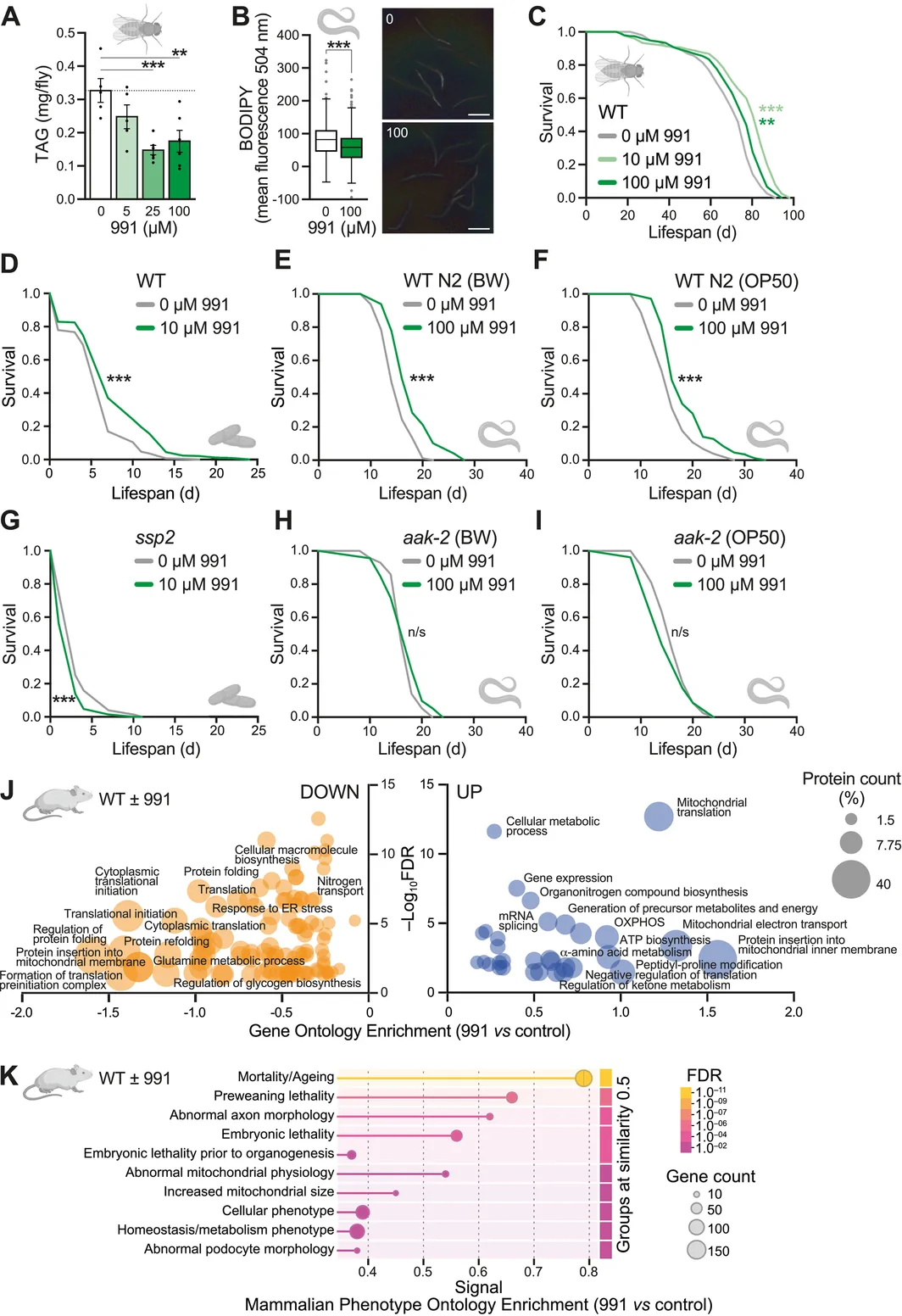
991 modulates metabolism and extends lifespan in *Drosophila*, *C. elegans* and *S. pombe*. (A) 991 treatment for 7 days in defined medium decreases whole body TAG levels of WT flies. Data are means ± SEM of *n* = 5–6 replicates per condition, each with *n* = 4 females per sample (one‐way ANOVA against the 0 μM DMSO control: 5 μM, *p* = 0.0378; 25 μM, *p* = 5.47 × 10^−4^; 100 μM, *p* = 0.0022). (B) Lipid analysis of worms by BODIPY staining. Fluorescence imaging of N2 WT worms comparing control (0 μM, *n* = 228) and 991‐treated (100 μM, *n* = 271). Scale bar, 500 μm. Data are box‐and‐whisker plots (Tukey), with statistical significance determined by an unpaired two‐tailed *t*‐test (*p* = 6.61 × 10^−7^). (C) 991 extends lifespan of WT *Drosophila* on defined medium (*n* ~ 150 females per condition; Log‐Rank test against 0 μM DMSO: 10 μM, *p* = 4.67 × 10^−11^; 100 μM, *p* = 0.0017). (D) 10 μM 991 extends chronological lifespan of WT *S. pombe* (*n* = 3 biological replicates, each with *n* = 2 technical repeats; Log‐Rank test, *p* = 1.18 × 10^−3^). (E, F) 991 extends lifespan of WT (N2) *C. elegans* on BW25113 bacteria (E, *p* = 2.95 × 10^−7^) and OP50 bacteria (F, *p* = 1.57 × 10^−5^) (*n* ~ 110 worms per condition; Log‐Rank test). (G) 10 μM 991 shortens chronological lifespan of AMPKα mutant (*ssp2*) *S. pombe* (*n* = 3 biological replicates, each with *n* = 2 technical repeats; Log‐Rank test; *p* = 1.77 × 10^−3^). (H, I) 100 μM 991 does not extend the lifespan of AMPKα mutant (*aak‐2*) worms on BW25113 bacteria (H, *p* = 0.2697) and OP50 bacteria (I, *p* = 0.1027) (*n* ~ 90–105 worms per condition; Log‐Rank test). (J, K) Treatment of WT mice with 991 in vivo induces a proteomic signature of pro‐longevity. Proteomics of liver tissue from WT male mice (*n* = 3 per condition) treated ±991‐loaded nanoparticles for 3 weeks. (J) Gene Ontology enrichment of biological processes showing terms with signal > 0.5 and/or significance *p* < 4 × 10^−5^. (K) Mammalian phenotype ontology enrichment (Monarch) from the STRING database with term grouping threshold > 0.5. ^n/s^ *p* > 0.05; **p < 0.01; ***p < 0.001.

Next, we focused on longevity, where AMPK is heavily implicated (Burkewitz et al. 2014, 2016). Dietary 991 treatment extended lifespan in WT flies (Figure 2C, Figure S2E). We did not observe changes in fly fecundity (Figure S2F). 991 also increased survival of WT *S. pombe* (Figure 2D), and WT N2 *C. elegans* (Figure 2E,F, Figure S2G,H). Higher doses of 991 shortened lifespan (Figure S2I,J), highlighting the importance of appropriate dosing for each organism and nutritional context. The worm lifespan effect was not bacterial strain‐dependent, as it was observed when the worms were cultured on both *E. coli* BW25113 (Figure 2E, Figure S2G) and OP50 (Figure 2F, Figure S2H). 991 did not have major effects on bacterial growth (Figure S2K,L), consistent with 991 acting directly on the worm host and not through the microbiota, as with metformin (Cabreiro et al. 2013). Since AMPKα‐null flies are not viable as adults, we therefore used yeast and worm AMPKα mutants to test drug specificity in vivo. Critically, 991 did not extend the lifespan of *ssp2* yeast; rather, a detrimental effect was observed, as the *ssp2* mutant cells are highly sensitive to any perturbations or stress (Schutt and Moseley 2017; Rodriguez‐Lopez et al. 2023) (Figure 2G), or *aak‐2* worms (Figure 2H,I, Figure S2M), indicating that the longevity effect was specifically through activation of AMPK by 991.

Altogether, we show that AMPK activation by 991 is evolutionarily conserved in yeast, worms and flies, allowing for the role of AMPK in the context of longevity to be directly pharmacologically tested within these tractable systems. We demonstrate that the direct AMPK activator 991 extends lifespan in *Drosophila*, *C. elegans* and *S. pombe*. These invertebrate and yeast models are key organisms in ageing research, as they lay the necessary groundwork for the successful translatability of pharmacological compounds targeting ageing processes to improve healthspan and lifespan in mammals (e.g., rapamycin, α‐ketoglutarate) (Harrison et al. 2009; Bjedov et al. 2010; Chin et al. 2014; Asadi Shahmirzadi et al. 2020).

### 2.3 Treatment of Mice With 991 In Vivo Induces a Proteomic Signature of Pro‐Longevity

To demonstrate the translational potential of our findings to mammals, we capitalised on a recently developed methodology for encapsulation of 991 into polymeric nanoparticles enabling delivery to mice as a pharmaceutical formulation that is biodegradable, bio‐compatible, and non‐toxic in vivo (Andreana et al. 2025). Unbiased proteome‐wide analysis of livers from WT mice (Figure 2J), as well as a muscular dystrophy model with reduced lifespan (Coley et al. 2016; Hart et al. 2024) (Dmd^mdx^, Figure S3A), treated ±991 for 3 weeks revealed a clear signature of AMPK activation (including increased ATP biosynthesis, mTOR inhibition and enhanced mitochondrial biogenesis). Furthermore, phenotypic ontology highlighted survival‐related processes as the most enriched category in response to 991 (Figure 2K, Figure S3B). Therefore, in vivo treatment of mice with 991 elicits a profile of protein translation and energy metabolism consistent with pro‐longevity interventions.

## 3 Discussion

Since AMPK is an attractive therapeutic target in the context of metabolic diseases, multiple pharmaceutical companies have developed promising second‐generation direct activators, including Merck (MK‐8722), Pfizer (PF‐739), Boehringer Ingelheim (BI‐9774), and Poxel (PXL‐770). Importantly, all these drugs bind in the same pocket as 991 (the ‘ADaM’ site) and directly activate AMPK via the same mechanism (Steinberg and Carling 2019). These second‐generation activators have improved bioavailability in mammalian systems compared to 991, without the requirement for nanoparticle encapsulation, and have already been demonstrated to activate AMPK in vivo in a range of mammalian studies (Table S1). These 991‐like direct AMPK activators have been extensively used in rodents for short‐term metabolic investigations, showing wide‐ranging improvements in physiological parameters and protection in diverse models of metabolic and age‐related diseases (Cokorinos et al. 2017; Myers et al. 2017; Grempler et al. 2018; Zhang et al. 2020; Gluais‐Dagorn et al. 2022; Penfold et al. 2023; Boehringer Ingelheim 2024). The 991‐like direct AMPK activator PXL‐770 improves glycemia, dyslipidemia, and insulin resistance in rodent models of metabolic syndrome, and reduces hepatic steatosis, inflammation, and fibrogenesis in a mouse model of metabolic dysfunction‐associated steatohepatitis (MASH—previously non‐alcoholic steatohepatitis, NASH) (Gluais‐Dagorn et al. 2022). Importantly, non‐human primates have been treated in vivo with MK‐8722 and PF‐739 for metabolic studies, focussed primarily on glucose homeostasis (Cokorinos et al. 2017; Myers et al. 2017), and PXL‐770 has been used in human clinical trials, showing good safety (Cusi et al. 2021; Fouqueray et al. 2021). Patients with metabolic dysfunction‐associated steatotic liver disease (MASLD—previously non‐alcoholic fatty liver disease, NAFLD) exhibit improved lipid profiles after 4 weeks of PXL‐770 treatment (Fouqueray et al. 2021).

In summary, our cross‐species study provides important proof‐of‐concept for direct AMPK activation as a pharmacological pro‐longevity target. The existing evidence from rodent, primate, and human studies confirming in vivo activation of AMPK by direct 991‐like molecules and metabolic effects indicates strong potential for translation to mammals, and that mammalian lifespan trials are now warranted.

### 3.1 Limitations of the Study

The present study demonstrates evolutionarily conserved life‐span extension by the direct AMPK activator 991 in *S. pombe*, *C. elegans* and *Drosophila*. Future efforts will focus on translating these results to mammalian systems. While our preliminary proteomic profiling data in mice are consistent with in vivo activation of AMPK upon nanoparticle delivery of 991, further mouse experiments are required. Longer‐term metabolic and longevity studies will involve formulation of the activator in the diet, rather than administration by injection or gavage as with short‐term interventions to date. Future mouse studies will likely be performed with 991‐like second‐generation activators, which exhibit improved bioavailability in mammalian systems compared to 991. Additional healthspan measures should be included to complement survival outcomes. Our genetic evidence showing lack of lifespan extension by 991 in AMPK‐null yeast and worms supports that the longevity effect is acting specifically via AMPK. Further work is required to elucidate the mechanism(s) underlying the lifespan extension downstream of direct pharmacological AMPK activation in vivo.

## 4 Materials and Methods

### 4.1 991 Synthesis and Storage

The compound 991 (MW 431.88) was synthesised by the company Selvita Ltd. as described (Xiao et al. 2013). Solutions (from a 0.1 M stock in DMSO) were prepared fresh prior to use. For mouse experiments, 991 was ordered from MedChemExpress (HY‐112769). Empty and 991‐loaded polymeric poly(lactic‐*co*‐glycolic) acid (PLGA) nanoparticles were prepared and physico‐chemically characterised as previously described (Andreana et al. 2025).

### 4.2 Sequence Alignment and Structural Modelling

Multiple sequence alignments for the AMPK α and β subunits were performed in T‐Coffee (Di Tommaso et al. 2011), and visualised using Jalview (Waterhouse et al. 2009). The following UniProt IDs were used: human, α2 P54646, β1 Q9Y478; *Drosophila*, AMPKα, O18645, alc (β), A1Z7Q8; *C. elegans* , aak‐2 (α) Q95ZQ4, aakb‐1 (β) Q9XUY2; *S. pombe*, ssp2 (α), O74536, amk2 (β) P78789. To visualise the substituted residues, homology modelling was performed using the SWISS‐MODEL server (Waterhouse et al. 2018), with the crystal structure of human AMPK α2β1γ1 (PDB: 4CFE) as the template structure.

### 4.3 *Drosophila* Cell Culture

For *Drosophila* cell culture, the S2R+ line (DGRC Stock 150; RRID:CVCL_Z831) was incubated at 25°C (ambient CO_2_) in Schneider's Medium (Gibco, 21720024) plus 10% heat‐inactivated FBS (Life Technologies, 16140‐071). For experiments, cells were seeded at 5 × 10^5^ per well in a 6‐well plate, and used the next day after changing to fresh medium 3 h prior to harvesting or treatment. For AMPK activation, cells were incubated with 991 (0.1–5 μM from a 0.1 M stock in DMSO), metformin (Sigma D150959; 5 mM from a 0.2 M stock in H_2_O), or phenformin (Sigma P7045; 1 mM from a 0.5 M stock in DMSO) for 1 h at 25°C prior to rapid lysis of the cells in situ.

### 4.4 *Drosophila* Husbandry and Lifespan Assays

For *Drosophila* experiments, *white Dahomey* (*w* ^*Dah*^) was used as the outbred wild‐type (WT) strain, maintained at 25°C, 65% humidity, 12 h:12 h light/dark cycles. Synchronised cohorts of flies were reared at constant density in bottles on yeast‐based medium (SYA), containing 5% w/v sucrose (granulated sugar), 10% w/v Brewer's yeast (MP Biomedicals 903312), 1.5% agar (Sigma A7002), supplemented with nipagin (Sigma H5501; 30 mL/L of 10% w/v nipagin in 95% EtOH) and propionic acid (0.3% v/v; Sigma P1386) as preservatives. After eclosion, flies were kept as a mixed population on fresh SYA for 48 h to allow maturation and mating, and then females were selected under mild CO_2_ anaesthesia into groups of 15 per vial. Drug treatments were started at this stage (= Day 2 of adulthood). 991 was administered either in SYA or a chemically defined (= holidic) diet (Piper et al. 2014), with the drug added from a 0.1 M stock in DMSO (final 0.1% v/v DMSO) when the medium was cooled to ~50°C–60°C. For *Drosophila* lifespans, flies were transferred without anaesthesia to fresh vials of medium every ~2–3 days, with deaths and censors scored (van Dam et al. 2020). Feeding rates were measured either using the automated FlyPAD system (Itskov et al. 2014), with the cumulative number of sips by individual flies assayed over 1 h. Alternatively, feeding rates were assessed based on behavioural observation of proboscis extension, scored blinded over 45 min, after 15 min of undisturbed observer presence (*n* = 10 vials per condition, each containing 5 flies). Fecundity was assayed as the number of eggs laid per female over a 24 h period counted blinded under a stereomicroscope (*n* = 10 vials per condition, each with 15 females per vial).

### 4.5 *C. elegans* Strains and Lifespan Assays

*C. elegans* N2 strain (WT) was obtained from the *Caenorhabditis* Genetics Centre (CGC, University of Minnesota), and strain GA1001 (*aak‐2* *(* *ok524* *)X*; AMPKα knock‐out mutant) was kindly provided by David Gems (UCL). Worms were maintained at 20°C on Nematode Growth Media (NGM) agar plates, seeded with either *E. coli* (either BW25113 or OP50) 3 days before worms were added. For experimental plates, molten NGM was supplemented with either DMSO as a control or 100 μM 991 (from a 0.1 M stock in DMSO, 1% v/v final).

For lifespans, worms were synchronised and transferred to experimental plates at larval stage 4 (L4). To prevent development of eggs, 5‐fluoro‐2′‐deoxyuridine (Thermo Scientific L16497.ME) was added to the plates at a 5 μM final concentration. Worms were scored manually every 2 days and were transferred to fresh plates on Days 4 and 8. Worms that went missing or showed bagging/vulva protrusion were censored.

### 4.6 Bacterial Growth Assays

Growth assays of *E. coli* strains BW25113 and OP50 were performed in clear flat‐bottom 96‐well plates with 200 μL liquid LB medium (Fisher BioReagents, BP9723‐2) per well, supplemented with either DMSO (1% v/v final) or 12.5–100 μM 991. In parallel, a control experiment was run ±50 mM metformin (from a 1 M stock in H_2_O). Before adding bacteria to the wells, overnight cultures were diluted 1:1000 in fresh LB medium. Bacterial growth was measured at 37°C every 25 min over ~24 h at OD_595_ (BioTek BioSpa 8 and Cytation 3).

### 4.7 *S. pombe* Strains, Growth and Chronological Lifespan Assays

Strains used in the study are *972* ^*−*^ (WT), *ssp2::kanMX6* (AMPKα deletion, constructed as previously described (Bahler et al. 1998)), and *h* ^*−*^ *ssp2‐13Myc::hphR* (Myc‐tagged AMPKα) (Schutt and Moseley 2017). Cells were grown in EMM2 minimal medium from overnight pre‐cultures, and treated in log growth phase (OD_600_ 0.5) with 10–100 μM of 991 compound as indicated, similarly to other pharmacological interventions (Rallis et al. 2017; Rodriguez‐Lopez et al. 2020). Chronological lifespan assays were conducted as previously described (Rallis et al. 2013, 2017). Briefly, cells were grown to OD_600_ 0.5 from overnight ‘mother’ cultures, treated with 991 or DMSO (0.1% v/v/final), and left to reach stationary phase at 32°C. From this time point (Day 0), samples from the cultures (*n* = 3 biological replicates, each with *n* = 2 technical repeats) were subjected to serial dilutions (adjusted across the time course) and assessment of colony forming units. Survival was calculated by normalising against Day 0.

### 4.8 Mouse Strains and 991 Treatment

WT (C57BL/6J; JAX #000664) and D2‐mdx (D2.B10‐Dmd^mdx^/J; JAX #013141) (Coley et al. 2016) mice were bred and used according to French legislation. The protocols were approved by the local ethical committee (CEEA‐010) of the University of Lyon and the French Ministry of Research. Experiments were conducted on 10–18 week old males fed a standard chow diet. For in vivo delivery, 991 was administered to isoflurane‐anaesthetised mice by intravenous (IV) injection of 991‐loaded PLGA nanoparticles in the tail vein (3.5 mg/kg of drug) 3 times per week (WT) or every other day (Dmd^mdx^) for 3 weeks. Control animals were administered the equivalent amount of empty PLGA nanoparticles. Tissues were collected, snap‐frozen in liquid nitrogen, and stored at −80°C.

### 4.9 Sample Preparation for Molecular Assays

#### 4.9.1 S2R+ Cells

S2R+ samples were prepared by rapid lysis in situ on the 6‐well plate. Following quick removal of the media, cells were briefly rinsed with 1 mL of ice‐cold PBS before addition of 250 μL ice‐cold HEPES lysis buffer (50 mM HEPES pH 7.5 at 4°C, 50 mM NaF, 5 mM Na_4_P_2_O_7_, 1 mM EDTA, 10% v/v glycerol, 1 mM DTT, 0.1 mM PMSF and 1 mM benzamidine), supplemented with 1% v/v Triton X‐100. Cells were scraped to resuspend, then transferred to pre‐chilled 1.5 mL tube, and centrifuged for 10 min at 15,000 rpm, 4°C. Cell lysate supernatants were transferred to fresh 1.5 mL tubes, and frozen on dry ice, before storage at −80°C until analysed. Active AMPK for use in dephosphorylation protection assays was prepared as above, except cells were harvested by slowly rinsing with ice‐cold PBS, then scraping into 250 μL ice‐cold HEPES lysis buffer (without Triton X‐100), and centrifuging for 5 min at 10,000 rpm, 4°C. The supernatant was discarded, and the cell pellet was resuspended by addition of 250 μL ice‐cold HEPES lysis buffer with aspiration. Protein concentrations were determined using the BCA assay (Pierce 23225).

#### 4.9.2 *Drosophila* Lysates

Cohorts of ~15–20 whole frozen flies per 2 mL tube were homogenised in 250 μL of ice‐cold homogenisation buffer (50 mM HEPES pH 7.5 at 4°C, 0.25 M sucrose, 50 mM NaF, 5 mM Na_4_P_2_O_7_, 1 mM EDTA, 1 mM DTT, 0.1 mM PMSF, 1 mM benzamidine) plus protease inhibitor cocktail (Complete Mini EDTA‐free, Roche 11836170001), using a stainless steel ball (5 mm diameter) in the TissueLyser II (Qiagen, 2 × 30 s at 30 Hz). Samples were maintained on ice for 2 min between rounds. After homogenisation, the metal ball was removed and Triton X‐100 added to a 1% v/v final concentration. Samples were incubated on ice for 20 min before centrifugation for 10 min at 13,000 rpm, 4°C. The lysate supernatant was partially purified using precipitation with polyethylene glycol 6000 (PEG_6000_, Merck 528877) at 6% final (Hardie et al. 2000). The pellet was resuspended in 100 μL of HEPES lysis buffer, and protein concentration was determined by the BCA assay (Pierce 23225). Lysates were either used directly for immunoprecipitation (IP), or aliquoted and stored at −80°C.

#### 4.9.3 *C. elegans* Lysates

*C. elegans* pellets (~5000 worms) were sonicated in 400 μL of pre‐chilled homogenisation buffer for 3 × 1 min at 4°C (Q700 sonicator, Qsonica; 100% amplitude). Triton X‐100 was added at a 1% (v/v) final concentration, then samples were mixed and incubated on ice for 20 min before centrifugation for 10 min at 13,000 rpm, 4°C. The lysate was removed to a fresh tube and partially purified by precipitation with PEG_6000_ (6% final). Samples were incubated on ice for 10 min prior to centrifugation for 10 min at 13,000 rpm, 4°C. and the pellet was then resuspended in 50 μL of HEPES lysis buffer.

#### 4.9.4 IP of *Drosophila* AMPK

Typically, 50 μg of *Drosophila* lysate was incubated in triplicate with 1 μL of QSSM antibody (custom‐made by New England Peptides based on a published peptide; Pan and Hardie 2002) or sheep antibody (NEP pre‐bleed) and 20 μL of 50% w/v protein G Sepharose (Sigma P3296) in 150 μL HEPES lysis buffer for 2 h at 4°C with shaking (IKA VXR Vibrax). IPs were then washed 2× with 0.5 mL of HEPES lysis buffer containing 1% v/v Triton X‐100, and finally with 0.5 mL HEPES lysis buffer. All buffer was carefully removed prior to assay.

### 4.10 AMPK Kinase Activity Assay

AMPK kinase activity was assayed based on the SAMS peptide method with incorporation of radiolabelled ATP (Davies et al. 1989; Woods et al. 1996). SAMS peptide (HMRSAMSGLHLVKRR sequence) was synthesised by Pepceuticals Ltd. *Drosophila* and *C. elegans* AMPK was assayed from either 6% PEG_6000_ lysates (5 μg protein) or from IP samples. Direct or allosteric (containing 991, phenformin or metformin) activity assays were performed in duplicate for 20 min at 30°C with agitation. The final volume of 25 μL contained 200 μM SAMS peptide, 200 μM AMP, 5 mM MgCl_2_ and 200 μM [γ^32^P]ATP (200–500 cpm/pmol; Perkin Elmer NEG502Z001MC) in assay buffer (50 mM HEPES buffer pH 7.5, 1 mM EDTA, 10% (v/v) glycerol) with 0.5% v/v Triton X‐100. After incubation, 20 μL aliquots were removed and spotted onto 1 cm^2^ squares of phosphocellulose paper (P81, Whatman). The squares were washed in 1% v/v orthophosphoric acid for ~20 min with 3× changes, then air dried before transferring to a scintillation tube containing 2 mL of EcoScint A solution (National Diagnostics, LS‐273) for liquid scintillation counting (Tricarb, Perkin Elmer). For dephosphorylation assays, active S2R+ AMPK IP samples were washed with 500 μL of 50 mM HEPES buffer pH 7.5, to ensure removal of all protein phosphatase inhibitors. The buffer was then carefully removed and the IP sample was then incubated in a 10 μL reaction with recombinant protein phosphatase 2C (PP2C, pre‐titred to give ~80%–90% reduction in AMPK activity) with 50 mM HEPES buffer pH 7.5, 5 mM MgCl_2_ and incubated ±991 for 10 min at 30°C. The immune complex was precipitated by brief centrifugation and washed twice with 500 μL HEPES lysis buffer, before analysis by the SAMS assay.

### 4.11 Immunoblotting

#### 4.11.1 Drosophila

For S2R+ lysates or whole fly homogenate 6% PEG_6000_ lysates, 30 μg protein was loaded per lane in Laemmli buffer onto a 4%–20% Criterion TGX Precast Midi Protein Gel (Bio‐Rad, 5671093) and run by SDS PAGE, before transferring to PVDF membrane (Millipore Immobilon‐FL, IPFL00010). Membranes were blocked with Odyssey Blocking Buffer (TBS; LI‐COR Biosciences, 927‐50100), then probed against phospho‐AMPKα (Cell Signaling Technology, 2535S; 1:1000), followed by goat anti‐rabbit IRDye 800CW secondary antibody (LI‐COR Biosciences, 926‐32211; 1:10,000). As a loading control, blots were probed for actin (Abcam, ab8224; 1:1000) or total AMPKα (α1 + α2, Abcam, ab80039; 1:1000), followed by donkey anti‐mouse IRDye 680LT secondary antibody (LI‐COR Biosciences, 926‐68022; 1:10,000). All antibodies were diluted in 1× TBS, 0.05% v/v TWEEN‐20 containing 5% skimmed milk powder. Blots were imaged using the LI‐COR Odyssey CLx, system, and quantified in Image Studio (v.5.2).

#### 4.11.2 *C. elegans*

Synchronised L4 worms were transferred to experimental plates. On Day 2 of adulthood, 75 worms were collected from experimental plates with M9 buffer and washed twice. Worms were lysed in 50 μL 1× Laemmli buffer (Bio‐Rad, 1610737) supplemented with 20 mM DTT by incubating at 95°C for 5 min, then centrifuging for 5 min at 1400 rpm, RT, followed by 5 min of sonication (100% amplitude, Qsonica). Next, 30 μL samples were run on a precast 4%–20% gel (Bio‐Rad, 5671093) and transferred onto a nitrocellulose membrane (Bio‐Rad, 1704159). The membrane was stained with Ponceau S and imaged to assess total protein, then blocked with 5% BSA (Millipore, 81‐065‐1) in 1 × PBS, 0.02% v/v TWEEN‐20 at room temperature for 1 h. Immunoblotting was done with phospho‐AMPKα (Cell Signaling Technology, 2535S; 1:1000), followed by anti‐rabbit IgG (Cell Signaling Technology, 7074S; 1:5000). Quantification was done in ImageJ (v.2.14.0). Samples were normalised to the amount of protein per sample (Ponceau S staining).

#### 4.11.3 S. pombe

Fission yeast western blots were conducted as previously described (Alao et al. 2020). For protein preparations, cells were diluted in RIPA buffer supplemented with protease (PMSF) and phosphatase inhibitors (Halt cocktail, EDTA‐free; 78441; Thermo Scientific) together with glass beads, and lysed in a Fastprep‐24 5G Homogeniser (MP Biomedicals, 40 s at 6.0 m/s using the QuickPrep adapter). Immunoblotting was done with phospho‐AMPKα (Thr172) rabbit antibody (D4D6D, Cell Signaling Technology, 50081; 1:1000) and anti‐Myc mouse antibody (9E10, Santa Cruz Biotechnology, sc‐40; 1:1000), followed by HRP‐conjugated anti‐rabbit or anti‐mouse secondary antibodies (Abcam AB205718 and ab205719; 1:2000). Quantification was done in ImageJ (v1.54i). Samples were normalised to the amount of protein per sample (Ponceau S staining).

### 4.12 Metabolic Analysis

#### 4.12.1 Drosophila

Whole fly body triglyceride (TAG) levels were measured as previously described (van Dam et al. 2020). Briefly, cohorts of *n* = 4 frozen flies per sample (~5–6 replicates per condition) were homogenised in 250 μL PBS containing 0.05% v/v TWEEN‐20 using a TissueLyser II (Qiagen, 1 min at 30 Hz). Homogenates were incubated at 70°C for 5 min before centrifugation for 1 min at 5000 rpm, 4°C. The supernatants were transferred to a fresh microfuge tube and spun again for 3 min at 14,000 rpm, 4°C, then decanted to a new tube. 5 μL aliquots of samples or standard (Thermo TR22923) were loaded onto a 96‐well plate, overlaid with 200 μL of Infinity Triglycerides reagent (Thermo TR22421), and incubated for 5 min at 37°C before reading the absorbance at OD_540_ in a spectrophotometer (FLUOstar Omega, BMG Labtech).

Whole body fly glycogen levels were measured as previously described (Lennicke et al. 2025). Briefly, groups of *n* = 4 frozen flies per sample (~6 replicates per condition) were weighed and placed in 2 mL tubes with glass beads, and homogenised in 250 μL of saturated Na_2_SO_4_ solution using a TissueLyser II (Qiagen, 1 min at 30 Hz). Homogenates were centrifuged for 1 min at 10,000 rpm, RT. Samples (80 μL) were transferred to microfuge tubes containing 800 μL of chloroform:methanol solution, mixed, then centrifuged for 5 min at 10,000 rpm, RT. The supernatants were removed, and 1 mL of anthrone solution (Sigma 319899, 71 mg anthrone in 50 mL of 70% H_2_SO_4_) was added to the glycogen pellet (or glycogen standards prepared in parallel), and incubated at 90°C for 20 min with gentle mixing, before loading onto a 96‐well plate and reading the absorbance at OD_620_ in a spectrophotometer (FLUOstar Omega; BMG Labtech).

#### 4.12.2 *C. elegans*

For lipid staining, worms were synchronised and added to experimental plates at L4. On Day 2 of adulthood, worms were collected from plates with M9 buffer and washed twice. Worms were incubated for 1 h in 120 μL MRWP buffer (160 mM KCl, 40 mM NaCl, 14 mM EGTA, 1 mM Spermidine‐HCl, 0.4 mM Spermine, 30 mM Na‐PIPES, 2% PFA and 0.2% β‐mercaptoethanol in milliQ‐H_2_O). BODIPY dye (Thermo Fisher Scientific, DA3922; diluted 1:1000 in M9 buffer) was added, and worms were incubated for 1 h in the dark. Worms were washed twice with M9 and imaged (Zeiss Axio Zoom.V16, 25× magnification). Fluorescent intensity was analysed in ImageJ (v.2.14.0) and normalised for background signal.

#### 4.12.3 Mouse Proteomics

Frozen liver tissue (~12–33 mg wet weight) from mice treated ±991 was ground with a micro pestle, then lysed in chilled 8 M urea buffer, supplemented with protease inhibitor tablets (Roche, 4693132001) in tubes containing ceramic beads (Precellys Evolution Touch Homogeniser, Bertin Technologies; 3 × 30 s at 6500 rpm, 9°C), followed by sonication (Bioruptor, Diagenode; 10 min at 4°C). Lysates were digested overnight with trypsin and then purified by styrene‐divinylbenzene reversed‐phase sulfonate (SDB‐RPS) stage tip purification.

Samples were analysed by data‐independent acquisition mass spectrometry (DIA‐MS) on an Orbitrap Exploris 480 mass spectrometer coupled to a Vanquish Neo UHPLC system in direct flow set‐up (Thermo Scientific). Samples were loaded onto a 2 cm PepMap pre‐column (#164946) with a flow of 5 μL/min before reverse‐flushing onto an Aurora Ultimate 25 cm column (IonOpticks). Peptides were chromatographically separated with an initial flow rate of 400 nL/min and gradient of 4% B (0.1% formic acid in 80% acetonitrile) up to 8% in 3 min. The flow rate was then reduced to 300 nL/min and B increased to 35% in 77 min, followed by a column wash with 98% B at 400 nL/min and re‐equilibration to initial condition. MS1 scans were acquired over the range 399–1001 *m*/*z* at 15K resolution. Maximum injection time was set to 22 ms and the automatic gain control (AGC) target to 100%. MS2 scans ranging 400–1000 *m*/*z* were acquired at 30K resolution with a maximum injection time of 54 ms, an AGC target of 1000%, and in 30 × 20 *m*/*z* windows with an overlap of 1 *m*/*z*. All scans were stored as centroid.

For the gas‐phase fractionated library (Searle et al. 2020), a pool generated from all samples was analysed in 6 individual runs covering the range 400–1000 *m*/*z* in 100 *m*/*z* increments using identical LC settings as the samples. For each run, MS1 was acquired at 60K resolution with a maximum injection time of 98 ms and an AGC target of 100%. MS2 spectra were acquired at 30K resolution with a maximum injection time of 60 ms. Spectra were acquired in staggered 4 *m*/*z* windows, resulting in nominal 2 *m*/*z* windows after deconvolution using ProteoWizard (Chambers et al. 2012).

For sample processing, the gas‐phase fractionated library was built in DIA‐NN (v1.8.1) (Demichev et al. 2020) using a SwissProt mouse canonical database (UP589, downloaded 15/01/2024) with settings matching acquisition parameters. DIA‐NN was run with the additional command line prompts ‘—report‐lib‐info’ and ‘—relaxed‐prot‐inf’. Further output settings were: filtered at 0.01 FDR; N‐terminal methionine excision enabled; maximum number of missed cleavages = 1; minimum peptide length = 7; maximum peptide length = 30; minimum precursor *m*/*z* = 400; maximum precursor *m*/*z* = 1000; cysteine carbamidomethylation enabled as a fixed modification. DIA‐NN output was further filtered on library *q*‐value and global *q*‐value ≤ 0.01 and at least 2 unique peptides per protein using R (v4.1.3). Finally, label‐free quantitation (LFQ) values were calculated using the DIA‐NN R‐package. Analysis of results was performed in Perseus (v1.6.15) (Tyanova et al. 2016) by filtering for data completeness in replicate groups, FDR‐controlled *t*‐tests, and 1D enrichment analysis. For functional enrichment analysis, biological process Gene Ontology (GO) was performed using STRING (v12.0) on statistically significant up‐ and down‐regulated proteins with fold‐change > 1.1 for each genotype subset. For the Mammalian Phenotype Ontology, the up‐regulated proteins for each genotype were used with a similarity of terms threshold > 0.5 for grouping of terms. Similar results were obtained if both up‐ and down‐regulated datasets were combined for analysis.

### 4.13 Statistical Analysis

Lifespan curves were analysed by Log‐Rank test (see Table S2 for a summary of exact *n* numbers and *p* values). All other data were plotted and analysed by *t*‐test or one‐way ANOVA as appropriate using GraphPad Prism (v10.2.1), with detailed information provided in the corresponding figure legends. ^n/s^ *p* > 0.05; *p < 0.05; **p < 0.01; ***p < 0.001.

## Author Contributions

D.C., F.C. and H.M.C. conceived and designed the study. E.S., M.B., F.C.L., J.‐P.A., C.L., A.F., C.R., F.C. and H.M.C. performed the research. B.C., G.L., R.M. and G.J. developed the 991‐loaded PLGA nanoparticles and generated the mouse samples. J.R.W. carried out the structural modelling. S.J.G. provided supervision. F.C. and H.M.C. wrote the manuscript, with input from all the authors.

## Funding

This work was funded by the Medical Research Council to D.C. (MC‐M302‐M3410) and H.M.C. (MC‐M302‐M3450). E.d.S. was supported by an ERDA award from the Institute of Clinical Sciences, Imperial College London. F.C. received funding from the Wellcome Trust/Royal Society (102532/Z/12/Z and 102531/Z/13/A), the DFG German Research Foundation (EXC 2030‐390661388), and the Center for Molecular Medicine Cologne (Individual Project Funding Program IPFP‐B02). Proteomics analysis was performed by the CECAD Proteomics Facility, supported by a DFG large instrument grant (INST 1856/71‐1 FUGG). C.R. was supported by research grants from the Royal Society (RGS\R1\201348), the Biotechnology and Biological Sciences Research Council (BB/V006916/1 and BB/V006916/2), and the Medical Research Council (MR/W001462/1 and MR/W001462/2). B.C., G.L., R.M. and G.J. were supported by a grant from Fondation Maladies Rares and AFM Téléthon (N°POCs(2023)‐121501).

## Conflicts of Interest

The authors declare no conflicts of interest.

## Supporting information


**Figure S1:** Direct pharmacological activation by 991 of AMPK from *Drosophila*, *C. elegans* and *S. pombe* in cell culture and in vivo. (A, B) Multiple sequence alignment of the AMPK α (A) and β (B) subunits from human (*Hs*), *D. melanogaster* (*Dm*), *C. elegans* (*Ce*) and *S. pombe* (*Sp*). Sequence identity across the species is indicated by colour coding. The conserved threonine residue in AMPKα (T172/184/243/189, respectively) essential for kinase activity is highlighted in red. The serine residue in AMPKβ (S108/185/91/130, respectively) that promotes binding of 991 is highlighted in magenta. (C) Full *Drosophila* western blot images corresponding to Figure 1H. (D) In vivo administration of 991 to *Drosophila*, dietarily in SYA medium for 7 days, does not activate AMPK (unlike in defined medium, see Figure 1I) as assessed by kinase assay (*n* = 2 ± range; one‐way ANOVA against the 0 μM DMSO control: 5 μM, *p* = 0.8886; 25 μM, *p* = 0.0050; 100 μM, *p* = 0.1117; 250 μM, *p* = 0.7535; 500 μM, *p* = 0.5574). (E) Western blot images corresponding to the quantification in Figure 1J. (F) Activation of OP50‐fed *C. elegans* AMPK by 991 in vivo based on western blotting of phospho‐AMPKα normalised to total protein (*n* = 3 ± SEM; *p* = 0.0413, two‐tailed unpaired *t*‐test). (G) Full *C. elegans* western blot images corresponding to Figure 1K and Figure S1E. (H) Full *S. pombe* western blot images corresponding to Figure 1L. n/s, *p* > 0.05; *p < 0.05; **p < 0.01.
**Figure S2:** 991 modulates metabolism and extends lifespan in *Drosophila*, *C. elegans* and *S. pombe*. (A) 991 treatment for 7 days does not affect glycogen levels in WT flies. Data are means ± SEM of *n* = 6 replicates, each containing *n* = 4 flies (one‐way ANOVA against the 0 μM DMSO control: 5 μM, *p* = 0.3443; 25 μM, *p* = 0.4939; 100 μM, *p* = 0.4263). (B, C) 991 does not affect feeding rates of WT flies. (B) Number of sips measured over 1 h by the automated FlyPAD system. Data are means ± SEM of *n* = 6–8 individual flies per condition. Left: naïve 7‐day old females, exposed to 991 for the first time (one‐way ANOVA against the H_2_O control: DMSO, *p* = 0.4686; 10 μM 991, *p* = 0.5650; 100 μM 991, *p* = 0.3475). Right: females pre‐treated with 991 for 7 days (one‐way ANOVA against the 0 μM DMSO control: 10 μM 991, *p* = 0.9235; 100 μM 991, *p* = 0.9619). (C) Behavioural observation of proboscis extension over 45 min, expressed as a proportion of total flies. Left: Each timepoint is the average of *n* = 10 vials, each containing *n* = 5 flies. Right: Summary across the time course. Data are means ± SEM of *n* = 10 time points (one‐way ANOVA against the 0 μM DMSO control: 10 μM 991, *p* = 0.4922; 25 μM 991, *p* = 0.1538; 100 μM 991, *p* = 0.4922). (D) Validation of the BODIPY lipid staining in *C. elegans* . Fluorescence imaging of N2 WT worms comparing OP50‐fed controls (*n* = 290) and starved for 24 h (*n* = 239). Scale bar, 500 μm. Data are box‐and‐whisker plots (Tukey), with statistical significance determined by an unpaired two‐tailed *t*‐test (*p* = 1.99 × 10^−8^). (E) Control dose response survival curves of WT females on defined medium supplemented with 0.01%–2% (v/v) DMSO, as a vehicle for 991, establishing that 0.1% is well below the threshold of toxicity (*n* ~ 150 per condition; Log‐Rank test against the 0% H_2_O control: 0.01%, *p* = 0.4039; 0.1%, *p* = 0.5161; 0.5%, *p* = 0.5082; 1%, *p* = 0.0213; 2%, *p* = 1.08 × 10^−35^). (F) Fly fecundity is not affected by 991, measured over 24 h after 7 days of treatment. Data are means ± SD of *n* = 10 vials, each with 15 females (one‐way ANOVA against the 0% DMSO control: 10 μM, *p* = 0.4348; 25 μM, *p* = 0.4083; 100 μM, *p* = 0.1684). (G, H) 991 extends lifespan of WT (N2) *C. elegans* on BW25113 bacteria (G, *p* < 0.0001) and OP50 bacteria (H, *p* < 0.001). Independent biological repeats, with *n* ~ 80–100 worms per condition, analysed by Log‐Rank test. (I) 250 μM 991 in defined medium shortens WT fly lifespan (*n* ~ 225 per condition; *p* = 1.03 × 10^−16^, Log‐Rank test). (J) 100 μM 991 shortens chronological lifespan in WT *S*. *pombe* (Log‐Rank test; *p* = 3.49 × 10^−5^). (K, L) Bacterial dose–response growth curves of *E. coli* BW25113 (left) and OP50 (right) treated with 12.5–100 μM 991 (K) or 50 mM metformin (L). Data are plotted as the mean ± SD of *n* = 3 replicates. (M) 991 does not extend the lifespan of AMPKα mutant (*aak‐2*) worms on OP50 bacteria. Independent biological repeat, with *n* ~ 80 worms per condition, analysed by Log‐Rank test (*p* > 0.05). n/s, *p* > 0.05; *p < 0.05; ***p < 0.001.
**Figure S3:** Treatment of a muscular dystrophy mouse model (Dmd^mdx^) with 991 in vivo. (A) Proteomics of liver tissue from Dmd^mdx^ male mice (*n* = 4 per condition) treated ±991‐loaded nanoparticles for 3 weeks. Gene Ontology enrichment of biological processes showing terms with signal > 0.3 and/or significance *p* < 6 × 10^−5^ for down‐regulated proteins and signal > 0.5 and/or significance *p* < 5 × 10^−5^ for up‐regulated proteins. (B) Mammalian phenotype ontology enrichment (Monarch) from the STRING database with term grouping threshold > 0.7.
**Table S1:** Summary of metabolic effects upon in vivo treatment of rodents with direct AMPK activators.
**Table S2:** Summary of survival data for *Drosophila* and *C. elegans* lifespans.

## Data Availability

The proteomics datasets have been deposited to the ProteomeXchange Consortium via the PRIDE partner repository with the accession number (PXD065733). All other data supporting the findings from this study are available within the manuscript and its Supporting Information, or from the corresponding authors upon reasonable request.
