# Supplementary material for: Direct Pharmacological Activation of AMPK Extends Lifespan in Yeast, Worms and Flies
Source: Aging Cell. 2026 Sep 29;25(10):e70721. doi: 10.1111/acel.70721 (PMC13623501; doi:10.1111/acel.70721)
Supplement: Supplementary file 1 [file ACEL-25-e70721-s001.pdf]

# Supplementary Figure 1

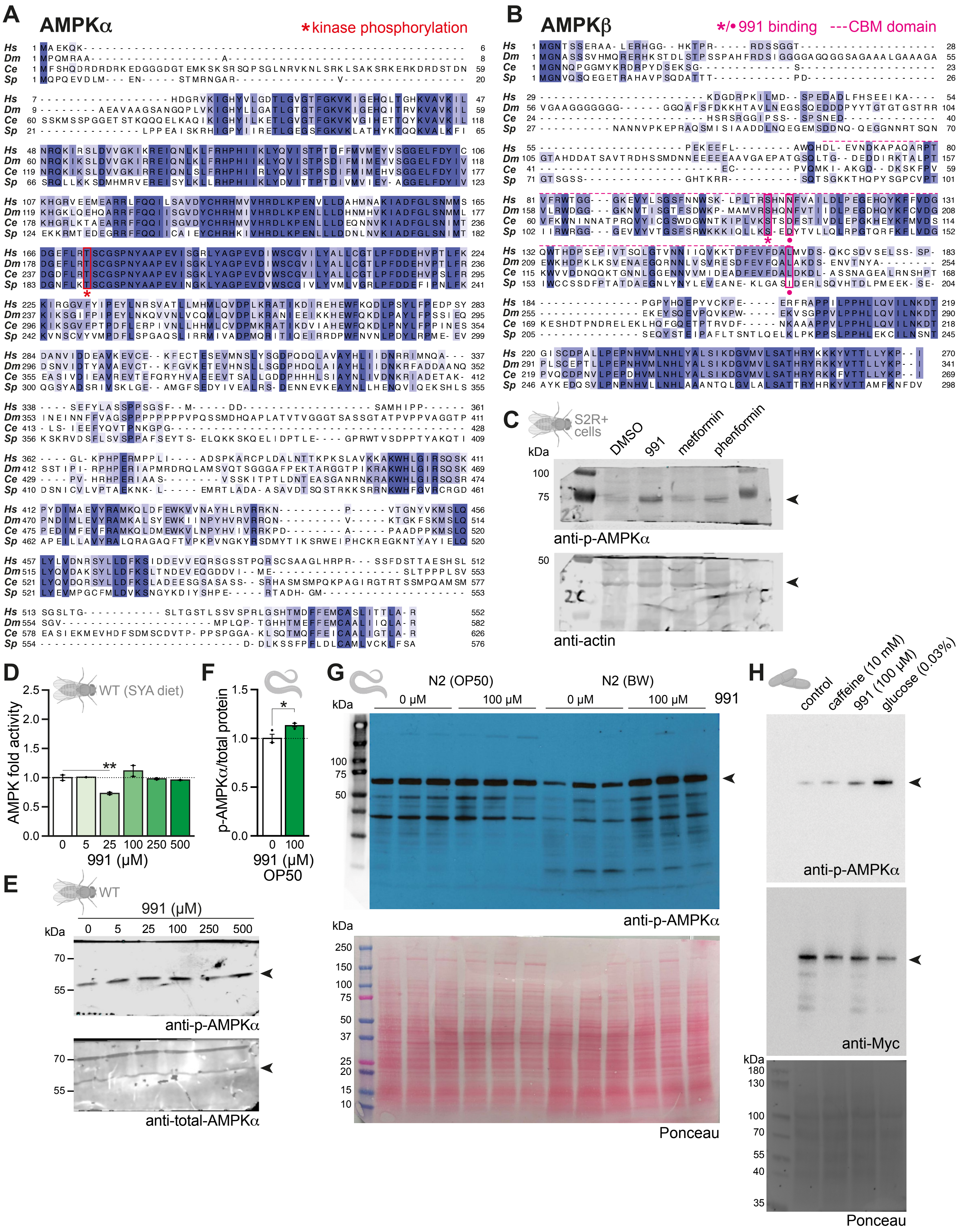

# Supplementary Figure 2

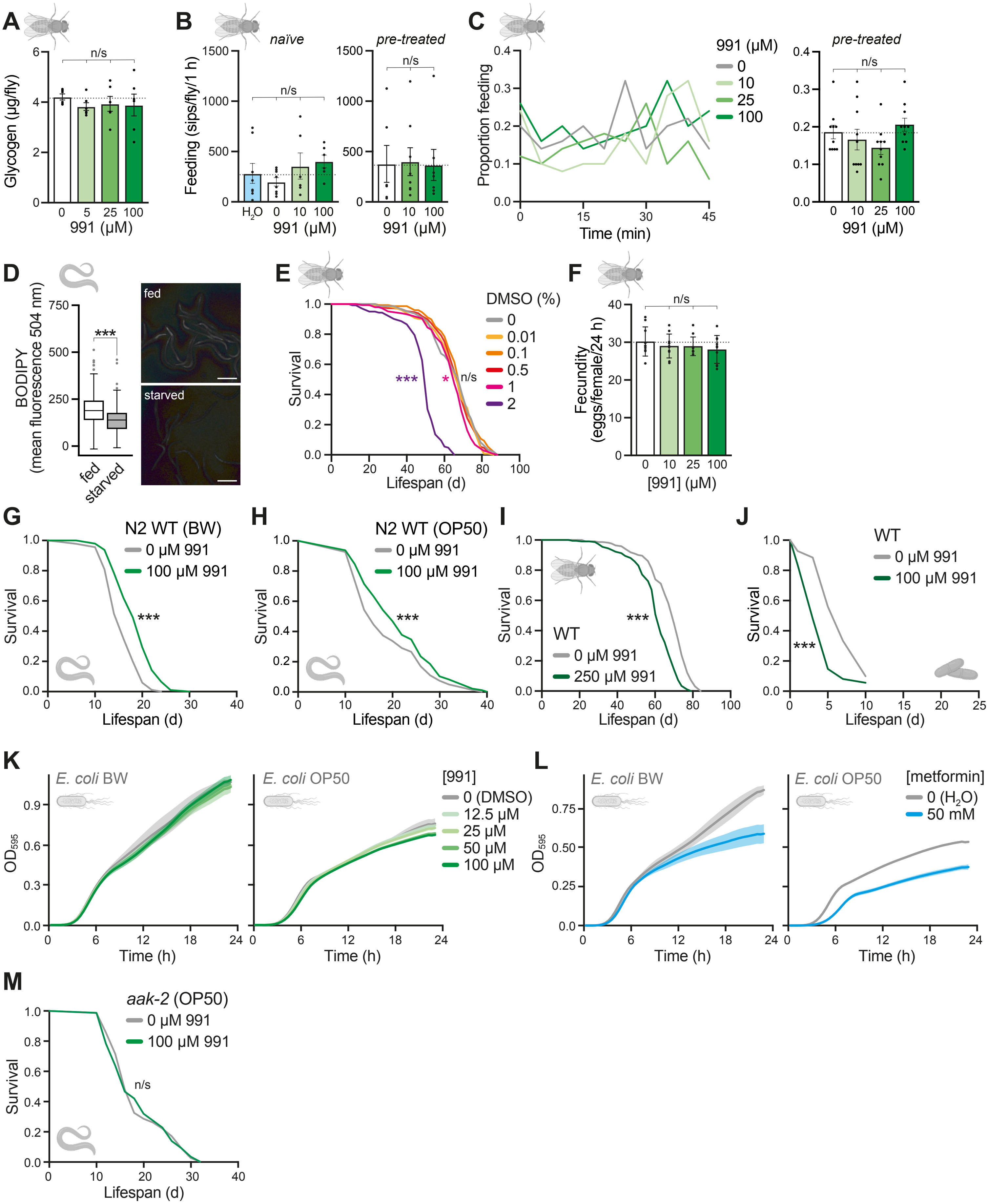

# Supplementary Figure 3

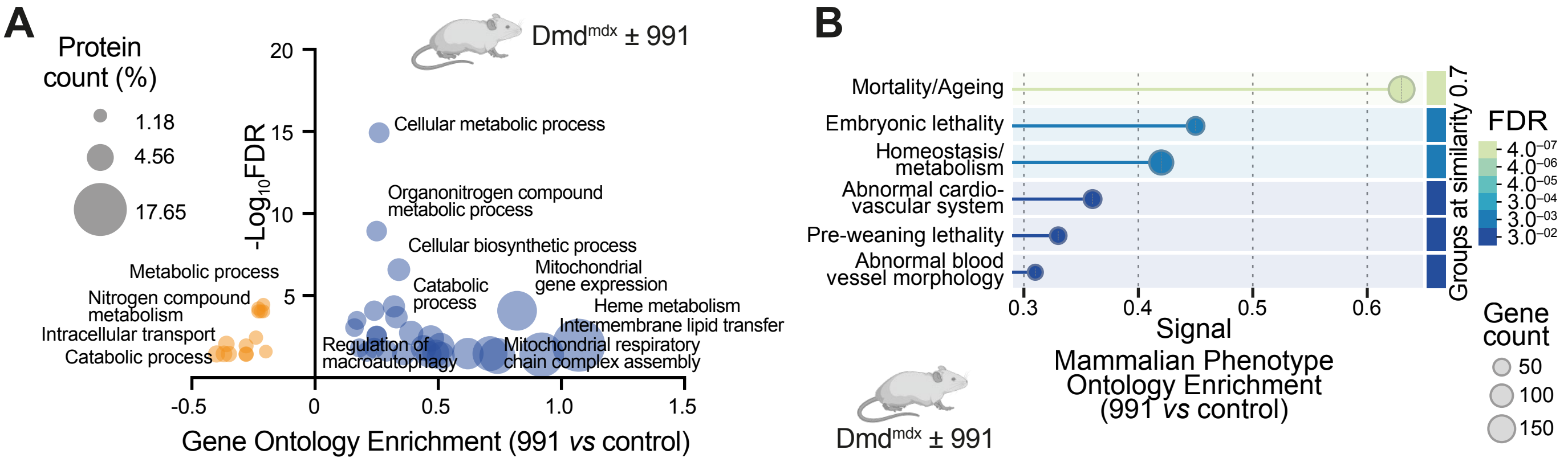

**SUPPLEMENTARY FIGURE LEGENDS**

**Figure S1 |** Direct pharmacological activation by 991 of AMPK from *Drosophila*, *C. elegans* and *S. pombe* in cell culture and *in vivo*. A-B) Multiple sequence alignment of the AMPK α (A) and β (B) subunits from human (*Hs*), *D. melanogaster* (*Dm*), *C. elegans* (*Ce*) and *S. pombe* (*Sp*). Sequence identity across the species is indicated by colour coding. The conserved threonine residue in AMPKα (T172/184/243/189 respectively) essential for kinase activity is highlighted in red. The serine residue in AMPKβ (S108/185/91/130 respectively) that promotes binding of 991 is highlighted in magenta. C) Full *Drosophila* western blot images corresponding to Figure 1H. D) *In vivo* administration of 991 to *Drosophila*, dietarily in SYA medium for 7 days, does not activate AMPK (unlike in defined medium, see Figure 1I) as assessed by kinase assay (n=2 ±range; one-way ANOVA against the 0 μM DMSO control: 5 μM, p=0.8886; 25 μM, p=0.0050; 100 μM, p=0.1117; 250 μM, p=0.7535; 500 μM, p=0.5574). E) Western blot images corresponding to the quantification in Figure 1J. F) Activation of OP50-fed *C. elegans* AMPK by 991 *in vivo* based on western blotting of phospho-AMPKα normalised to total protein (n=3 ±SEM; p=0.0413, two-tailed unpaired t-test). G) Full *C. elegans* western blot images corresponding to Figure 1K and Figure S1E. H) Full *S. pombe* western blot images corresponding to Figure 1L. n/s, p>0.05; *, p<0.05; **, p<0.01.

**Figure S2 |** 991 modulates metabolism and extends lifespan in *Drosophila*, *C. elegans* and *S. pombe.* A) 991 treatment for 7 days does not affect glycogen levels in WT flies. Data are means ±SEM of n=6 replicates, each containing n=4 flies (one-way ANOVA against the 0 μM DMSO control: 5 μM, p=0.3443; 25 μM, p=0.4939; 100 μM, p=0.4263). B-C) 991 does not affect feeding rates of WT flies. (B) Number of sips measured over 1 h by the automated FlyPAD system. Data are means ±SEM of n=6-8 individual flies per condition. Left: naïve 7-day old females, exposed to 991 for the first time (one-way ANOVA against the $H_2O$ control: DMSO, p=0.4686; 10 μM 991, p=0.5650; 100 μM 991, p=0.3475). Right: females pre-treated with 991 for 7 days (one-way ANOVA against the 0 μM DMSO control: 10 μM 991, p=0.9235; 100 μM 991, p=0.9619). (C) Behavioural observation of proboscis extension over 45 min, expressed as a proportion of total flies. Left: Each timepoint is the average of n=10 vials, each containing n=5 flies. Right: Summary across the time course. Data are means ±SEM of n=10 time points (one-way ANOVA against the 0 μM DMSO control: 10 μM 991, p=0.4922; 25 μM 991, p=0.1538; 100 μM 991, p=0.4922). D) Validation of the BODIPY lipid staining in *C. elegans*. Fluorescence imaging of N2 WT worms

comparing OP50-fed controls (n=290) and starved for 24 h (n=239). Scale bar, 500 μm. Data are box-and-whisker plots (Tukey), with statistical significance determined by an unpaired two-tailed t-test ($p=1.99x10^{-8}$). E) Control dose response survival curves of WT females on defined medium supplemented with 0.01-2% (v/v) DMSO, as a vehicle for 991, establishing that 0.1% is well below the threshold of toxicity (n~150 per condition; Log-Rank test against the 0% $H_2O$ control: 0.01%, p=0.4039; 0.1%, p=0.5161; 0.5%, p=0.5082; 1%, p=0.0213; 2%, $p=1.08x10^{-35}$). F) Fly fecundity is not affected by 991, measured over 24 h after 7 days of treatment. Data are means ±SD of n=10 vials, each with 15 females (one-way ANOVA against the 0% DMSO control: 10 μM, p=0.4348; 25 μM, p=0.4083; 100 μM, p=0.1684). G-H) 991 extends lifespan of WT (N2) *C. elegans* on BW25113 bacteria (G, p<0.0001) and OP50 bacteria (H, p<0.001). Independent biological repeats, with n~80-100 worms per condition, analysed by Log-Rank test. I) 250 μM 991 in defined medium shortens WT fly lifespan (n~225 per condition; $p=1.03x10^{-16}$, Log-Rank test). J) 100 μM 991 shortens chronological lifespan in WT *S. pombe* (Log-Rank test; $p=3.49x10^{-5}$). K-L) Bacterial dose-response growth curves of *E. coli* BW25113 (left) and OP50 (right) treated with 12.5-100 μM 991 (K) or 50 mM metformin (L). Data are plotted as the mean ±SD of n=3 replicates. M) 991 does not extend the lifespan of AMPKα mutant (*aak-2*) worms on OP50 bacteria. Independent biological repeat, with n~80 worms per condition, analysed by Log-Rank test (p>0.05). n/s, p>0.05; *, p<0.05; ***, p<0.001.

**Figure S3 |** Treatment of a muscular dystrophy mouse model ($Dmd^{mdx}$) with 991 *in vivo*. A) Proteomics of liver tissue from $Dmd^{mdx}$ male mice (n=4 per condition) treated ±991-loaded nanoparticles for 3 weeks. Gene ontology enrichment of biological processes showing terms with signal >0.3 and/or significance $p<6x10^{-5}$ for down-regulated proteins and signal >0.5 and/or significance $p<5x10^{-5}$ for up-regulated proteins. B) Mammalian phenotype ontology enrichment (Monarch) from the STRING database with term grouping threshold >0.7.

# SUPPLEMENTARY TABLES

**TABLE S1** | Summary of metabolic effects upon *in vivo* treatment of rodents with direct AMPK activators.

Overview of the literature on *in vivo* treatment (both acute and chronic) of mouse and rat models with 991-like AMPK activators, that bind within the same pocket at the interface of the α and β subunits.

| | Direct AMPK activator | Treatment duration | Rodent model | Metabolic/Physiological outcome | Study/Reference |
|---|---|---|---|---|---|
| 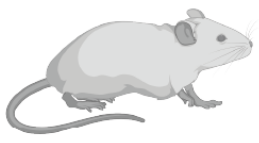 | PF-739 | 29/32 days | obese/diabetic mouse (DIO) | ↓ fat mass = body weight | Cokorinos et al. 2017 |
| | MK-8722 | 12 days | obese/diabetic mouse (db/db) | = body weight | Myers et al. 2017 |
| | | 28 days | obese/diabetic rat (ZDF) | = body weight | Myers et al. 2017 |
| | | 3 weeks | MASH/NASH mouse (STAM™) | = body weight | Zhang et al. 2020 |
| | BI-9774 | 6 weeks | obese/diabetic rat (ZDF) | ↓ body weight | Boehringer Ingelheim opnMe |
| | PXL-770 | 8 weeks | MASH/NASH mouse (AMLN diet) | ↓ body weight | Gluais-Dagorn et al. 2022 |
| **Heart** | PF-739 | acute | WT mouse | = glucose uptake ↑ [hexose-P] | Cokorinos et al. 2017 |
| | MK-8722 | 2 weeks | WT rat | = heart weight ↑ [glycogen] | Myers et al. 2017 |
| | | 1 month | WT rat | ↑ heart weight ↑ [glycogen] = cardiac function | Myers et al. 2017 |
| | | 14 days | obese/diabetic mouse (db/db) | ↑ heart weight | Myers et al. 2017 |
| | | chronic | obese/diabetic mouse (DIO) | ↑ heart weight | Myers et al. 2017 |
| | | 3 weeks | MASH/NASH mouse (STAM™) | = heart weight | Zhang et al. 2020 |
| | BI-9774 | acute | rat | protection in ischemia/reperfusion | Boehringer Ingelheim opnMe |
| | | 2 weeks | WT rat | ↓ heart rate ↓ inotropism ↑ heart weight ↑ [glycogen] | Boehringer Ingelheim opnMe |
| | | 6 weeks | obese/diabetic rat (ZDF) | ↓ heart rate ↓ inotropism = heart weight ↑ [glycogen] | Boehringer Ingelheim opnMe |
| **Skeletal muscle** | PF-739 | acute | WT mouse | ↑ glucose uptake ↑ [hexose-P] | Cokorinos et al. 2017 |
| | | acute | obese/diabetic mouse (DIO) | ↑ [glucose-6P, UDP-glucose, pyruvate, glycogen] | Cokorinos et al. 2017 |
| | | 32 days | obese/diabetic mouse (DIO) | ↑ [glycogen] | Cokorinos et al. 2017 |
| | MK-8722 | acute | WT mouse | ↑ glucose uptake | Myers et al. 2017 |
| | | acute | diabetic rat (GK) | ↑ [glucose-6P, glycogen, lactate] | Myers et al. 2017 |
| | | 2 weeks | WT mouse | ↑ Glut4 protein | Myers et al. 2017 |
| | | 1 month | WT rat | ↑ [glycogen] | Myers et al. 2017 |
| | BI-9774 | acute | rodent | ↑ glucose uptake ↑ [glycogen, lactate] | Boehringer Ingelheim opnMe |
| **Blood** | PF-739 | acute | WT mouse | ↓ [blood glucose] improved glucose tolerance | Cokorinos et al. 2017 |
| | | acute | obese/diabetic mouse (DIO) | ↓ [blood glucose] ↓ [plasma insulin] | Cokorinos et al. 2017 |
| | | acute | obese/diabetic mouse (db/db) | ↓ [blood glucose] | Cokorinos et al. 2017 |
| | | 30 days | obese/diabetic mouse (DIO) | ↓ [blood glucose] ↓ [plasma insulin] | Cokorinos et al. 2017 |
| | | 32 days | obese/diabetic mouse (DIO) | = [plasma TAG, lactate, FFA] ↑ [plasma β-hydroxybutyrate] | Cokorinos et al. 2017 |
| | MK-8722 | acute | WT mouse | ↓ [blood glucose] improved glucose tolerance ↓ [plasma insulin] | Myers et al. 2017 |
| | | acute | obese/diabetic mouse (DIO) | ↓ [blood glucose] improved glucose tolerance | Myers et al. 2017 |
| | | acute | obese/diabetic rat (ZDF) | ↓ [blood glucose] improved glucose tolerance ↓ [plasma insulin] | Myers et al. 2017 |
| | | acute | diabetic rat (GK) | ↓ [blood glucose] improved glucose tolerance | Myers et al. 2017 |
| | | 12 days | obese/diabetic mouse (db/db) | ↓ [blood glucose] | Myers et al. 2017 |
| | | 15 days | obese/diabetic mouse (DIO) | improved glucose tolerance | Myers et al. 2017 |
| | | 24 days | obese/diabetic mouse (DIO) | ↓ [plasma insulin] | Myers et al. 2017 |
| | | 28 days | obese/diabetic rat (ZDF) | ↓ [blood glucose] ↓ HbA1c | Myers et al. 2017 |
| | | 3 weeks | MASH/NASH mouse (STAM™) | = [blood glucose] = [plasma insulin, TAG, cholesterol] | Zhang et al. 2020 |
| | BI-9774 | acute | WT rat | ↓ [blood glucose] | Boehringer Ingelheim opnMe |
| | | acute | rodent | improved glucose tolerance | Boehringer Ingelheim opnMe |
| | | 6 weeks | obese/diabetic rat (ZDF) | ↓ [blood glucose] ↓ HbA1c | Boehringer Ingelheim opnMe |
| | | 28 days | obese/diabetic rat (ZDF) | ↓ [blood glucose] | Grempler et al. 2018 |
| | PXL-770 | 5 days | obese/diabetic mouse (ob/ob) | improved glucose tolerance | Gluais-Dagorn et al. 2022 |
| | | 5 weeks | obese/diabetic mouse (ob/ob) | ↓ HbA1c ↓ [plasma TAG, FFA, glycerol] | Gluais-Dagorn et al. 2022 |
| | | 6 weeks | obese/diabetic mouse (DIO) | ↑ insulin sensitivity | Gluais-Dagorn et al. 2022 |
| | | 8 weeks | MASH/NASH mouse (AMLN diet) | ↓ [plasma FFA, cholesterol] = [plasma TAG] = [blood glucose] | Gluais-Dagorn et al. 2022 |
| **Liver** | PF-739 | 32 days | obese/diabetic mouse (DIO) | ↓ [liver TAG] ↓ [plasma ALT] | Cokorinos et al. 2017 |
| | MK-8722 | 3 weeks | MASH/NASH mouse (STAM™) | ↓ [liver TAG] ↓ liver fibrosis | Zhang et al. 2020 |
| | | | | = liver weight = [plasma ALT, AST] | Zhang et al. 2020 |
| | PXL-770 | acute | WT mouse | ↓ hepatic *de novo* lipogenesis | Gluais-Dagorn et al. 2022 |
| | | 8 weeks | MASH/NASH mouse (AMLN diet) | ↓ liver steatosis ↓ [liver TAG] ↓ hepatocellular ballooning ↓ liver inflammation ↓ [plasma ALT, AST] ↓ liver weight ↓ liver fibrosis | Gluais-Dagorn et al. 2022 |
| **Pancreas** | MK-8722 | 28 days | obese/diabetic rat (ZDF) | ↑ [pancreatic insulin] | Myers et al. 2017 |
| **Prostate** | BI-9774 | 21 days | immunodeficient mouse (NSG) | ↓ prostate cancer xenograft growth | Penfold et al. 2023 |

ALT, alanine transaminase; AMLN, amylin liver NASH; AST, aspartate transaminase; DIO, diet-induced obesity; FFA, free fatty acids; GK, Goto-Kakizaki; HbA1c, hemoglobin A1c; MASH, metabolic dysfunction-associated steatohepatitis; NASH, non-alcoholic steatohepatitis; NSG, NOD scid gamma; TAG, triacyl-glycerides; ZDF, Zucker Diabetic Fatty.

**TABLE S2** | Summary of survival data for *Drosophila* and *C. elegans* lifespans.

| Figure | Organism | Genotype | Treatment | Deaths # | Censors # | Total # | Median survival (d) | Lifespan effect (Δ%) | Log-Rank p value |
|---|---|---|---|---|---|---|---|---|---|
| **Fig. 2C** | *Drosophila* | WT ($w^{Dah}$) | 0 µM 991 (DMSO) | 148 | 4 | 152 | 71.63 | | |
| | | | 10 µM 991 | 152 | 0 | 152 | 80.30 | 12.11 | 4.67E-11 |
| | | | 100 µM 991 | 149 | 3 | 152 | 75.05 | 4.78 | 0.0017 |
| | | | | | | 0 | | | |
| **Fig. 2E** | *C. elegans* | WT (N2), BW | 0 µM 991 (DMSO) | 95 | 14 | 109 | 13.80 | | |
| | | | 100 µM 991 | 98 | 7 | 105 | 15.93 | 15.43 | 2.95E-07 |
| | | | | | | 0 | | | |
| **Fig. 2F** | *C. elegans* | WT (N2), OP50 | 0 µM 991 (DMSO) | 102 | 11 | 113 | 14.35 | | |
| | | | 100 µM 991 | 99 | 16 | 115 | 15.88 | 10.66 | 1.57E-05 |
| | | | | | | 0 | | | |
| **Fig. 2H** | *C. elegans* | *aak-2* , BW | 0 µM 991 (DMSO) | 69 | 21 | 90 | 15.76 | | |
| | | | 100 µM 991 | 92 | 0 | 92 | 16.00 | 1.52 | 0.2697 |
| | | | | | | 0 | | | |
| **Fig. 2I** | *C. elegans* | *aak-2* , OP50 | 0 µM 991 (DMSO) | 88 | 17 | 105 | 15.16 | | |
| | | | 100 µM 991 | 92 | 13 | 105 | 13.28 | -12.40 | 0.1027 |
| **Fig. S2E** | *Drosophila* | WT ($w^{Dah}$) | 0% DMSO ($H_2O$) | 146 | 0 | 146 | 67.40 | | |
| | | | 0.01% DMSO | 132 | 16 | 148 | 66.18 | -1.82 | 0.4040 |
| | | | 0.1% DMSO | 149 | 0 | 149 | 67.38 | -0.04 | 0.5161 |
| | | | 0.5% DMSO | 149 | 1 | 150 | 66.40 | -1.48 | 0.5082 |
| | | | 1% DMSO | 148 | 3 | 151 | 64.70 | -4.01 | 0.0214 |
| | | | 2% DMSO | 132 | 13 | 145 | 49.28 | -26.89 | 1.08E-35 |
| | | | | | | 0 | | | |
| **Fig. S2G** | *C. elegans* | WT (N2), BW | 0 µM 991 (DMSO) | 86 | 6 | 92 | 14.20 | | |
| | | | 100 µM 991 | 92 | 8 | 100 | 17.87 | 25.85 | <0.0001 |
| **Fig. S2H** | *C. elegans* | WT (N2), OP50 | 0 µM 991 (DMSO) | 83 | 0 | 83 | 14.87 | | |
| | | | 100 µM 991 | 98 | 0 | 98 | 19.14 | 28.72 | <0.001 |
| **Fig. S2I** | *Drosophila* | WT ($w^{Dah}$) | 0 µM 991 (DMSO) | 222 | 3 | 225 | 68.9 | | |
| | | | 250 µM 991 | 208 | 13 | 221 | 60.375 | -12.37 | 1.03E-16 |
| **Fig. S2M** | *C. elegans* | *aak-2* , OP50 | 0 µM 991 (DMSO) | 77 | 0 | 77 | 15.84 | | |
| | | | 100 µM 991 | 88 | 0 | 88 | 15.60 | -1.52 | >0.05 |
